# A new class of signals for magnetobiology research

**DOI:** 10.1038/s41598-019-43984-z

**Published:** 2019-05-16

**Authors:** Leonardo Makinistian

**Affiliations:** Department of Physics and Instituto de Física Aplicada (INFAP), Universidad Nacional de San Luis-CONICET, San Luis, Argentina

**Keywords:** Bioenergetics, Molecular biophysics, Biological physics

## Abstract

The great majority of experimental and theoretical studies in magnetobiology explored and tried to explain bioeffects on organisms (ranging from bacteria to humans) upon exposure to variable (AC) magnetic fields (MF) with a pure sinusoidal waveform, typically combined with a static (DC) component. In this report, a new class of signals is presented and posed as a relevant candidate for research in magnetobiology. The proposed signals are derived within the classic theory of the precession of a magnetic moment in a DC + AC MF in a parallel configuration. They display a frequency modulation such that the phase change per unit time of the applied AC field is, at all times, identical to that of the precession of the magnetic moment to which the field was tuned (considering its gyromagnetic ratio). In other words, applied AC field and precession of the ‘engaged’ magnetic moment are phase-locked. These phase-locked frequency modulated (PLFM) signals are discussed in the context of current literature, and possible future experimental and theoretical developments are suggested.

## Introduction

Historically, sinusoids have been the paradigmatic signals for exploring the response of systems to oscillatory stimuli in all branches of the natural sciences, including magnetobiology. Indeed, the great majority of experimental and theoretical studies on the interaction of combinations of static (DC) and alternating (AC) extremely low frequency magnetic fields (ELF-MF) with living organisms (ranging from bacteria to humans) explored bioeffects upon exposure to pure sinusoids^1,2^. In this report, a new class of signals is presented and posed as a relevant candidate for research in magnetobiology.

The mathematical derivation of the signals proposed here is done within the frame of the phenomenon of Larmor precession of a magnetic moment; which has been proposed since the early 1990’s as a possible mechanism of transduction of weak DC and ELF-MF by living systems^3^. The model was particularly appealing because it avoided the strong criticisms^4^ that the Ion Cyclotron Resonance^5^ mechanism had received regarding unrealistically large trajectory radius and the effect of friction. Edmonds^3^ placed the transduction event inside an enzymatic cavity with a bound ion inside, and hypothesized that the precession of the ion would change the biochemical reactivity of the host enzyme. An explicit treatment of thermal fluctuations was done through a Langevin-Lorenz model^6–8^, quantitatively predicting that, for low enough viscosities, a sensitivity for fields in the *μ*T-range was possible. Later on, within the same formalism, Pilla *et al*.^9^ postulated that the binding kinetics of an ion to a hydrated macromolecule could be affected by a precessional motion of the bound water molecules. Larmor precession was also present in quantum mechanical models of a bound ion, where relatively short binding times due to thermal agitation where a manifest unresolved problem^6,10^. In contrast, back within a fully classic Lorenz-Langevin model, Muehsam and Pilla^11^ concluded that thermal noise does not break coherence of the Larmor precession during the binding lifetime, and that, in fact, it contributes to the effects of ELF-MF on biochemical reactivity^12^ (instead of hampering it).

More recently, Binhi^13^ presented a model of interaction also based on Larmor precession, posing that the maximum biological effect was to be expected as a result of those intervals of time in which precession is stopped (or almost stopped) by the transient cancellation of the DC field by a parallel AC field. Binhi and Prato analysed and further developed the model^14^, to finally extend it to include rotation of macromolecules into the picture of interaction^15^. While their model goes deeply into the details of how the first stage of transduction is eventually translated to an actual biological effect through downstream events, the signals presented here focus at that very first transduction stage. They aim at altering the dynamics of the precession in a stronger way than sinusoids, by producing longer periods of relatively “stopped” precession. Therefore, this signals could, presumably, be more effective than sinusoids to elicit biological effects.

## Phase-Locked Frequency Modulated (PLFM) Signals

We set out from the fundamental relations of the instantaneous torque *τ*(*t*) of a magnetic field ***B***(*t*) exerted over a magnetic moment ***m***(*t*), and that of the conservation of angular momentum, ${\boldsymbol{L}}(t)=\frac{{\boldsymbol{m}}(t)}{\gamma }$ (where *γ* is the so-called gyromagnetic ratio), to find the well known equation of motion for the magnetic moment:

1 $$\begin{array}{rcl}\tau (t) & = & {\boldsymbol{m}}(t)\times {\boldsymbol{B}}(t)\\ \tau (t) & = & \frac{d{\boldsymbol{L}}(t)}{dt}=\frac{1}{\gamma }\frac{d{\boldsymbol{m}}(t)}{dt}\end{array}\}\Rightarrow \frac{d{\boldsymbol{m}}(t)}{dt}=\gamma {\boldsymbol{m}}(t)\times {\boldsymbol{B}}(t)$$

**Figure 1:**
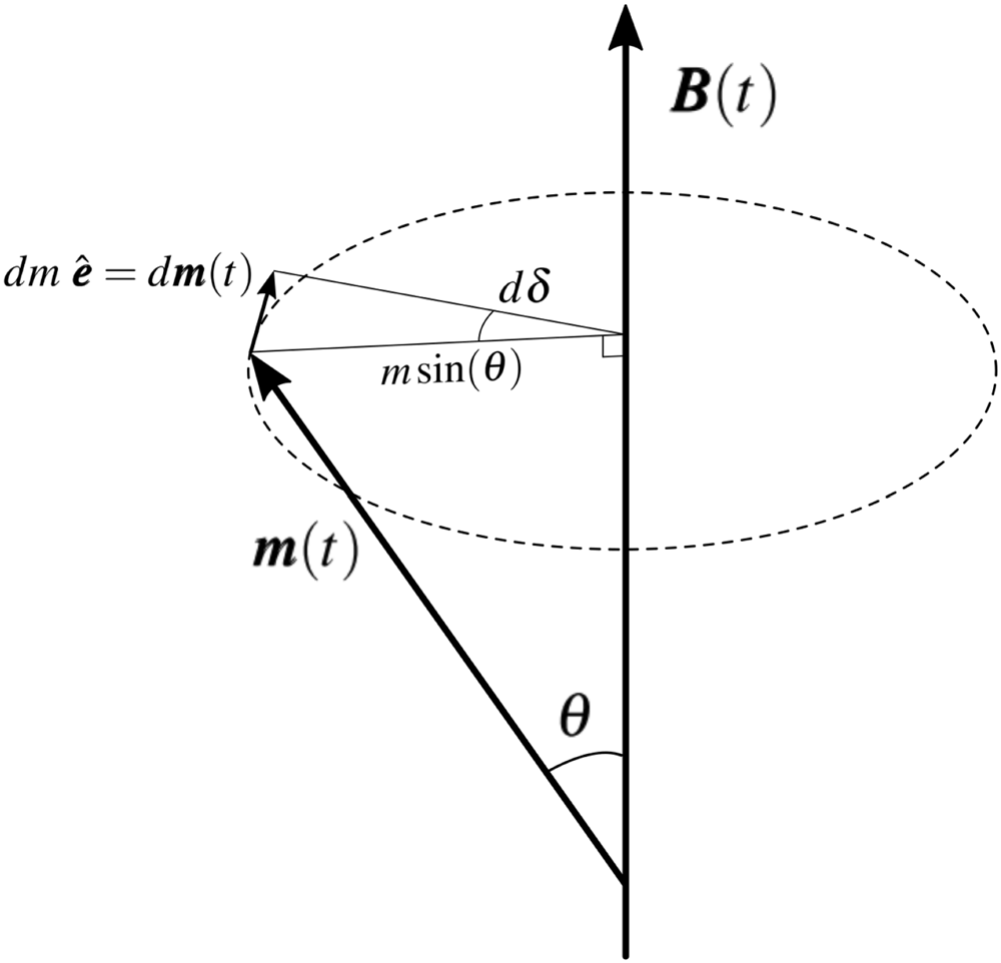
Vector relations for the derivation of the (instantaneous) Larmor frequency. *dδ* is the differential of the instantaneous phase of precession, $\hat{{\boldsymbol{e}}}$ is the unit vector perpendicular to the radius at all times, and *m* = |***m***(*t*)| is the absolute value of the magnetic moment. It is assumed here that *m* and *θ* (the angle of precession) both remain constant, and that the amplitude of the magnetic field is modulated as |***B***(*t*)| = *B*_*DC*_ + *B*_*AC*_(*t*), while its direction stays fixed (i.e., ***B***_*DC*_∥***B***_*AC*_).

Equation 1 equals the Landau-Lifshitz-Gilbert equation *without the dissipation term* (or, equivalently, the Bloch equation with T_1_ and T_2_ tending to infinity), that is, we hereafter assume negligible damping and relaxations. Careful consideration of this crucial point will be given below, in the Discussion. Now, from the vector relations shown in Fig. 1, where *m* = |***m***(*t*)|, it is immediate that $d{\boldsymbol{m}}(t)=d\delta \,m\,\sin \,(\theta )\hat{{\boldsymbol{e}}}$; and differentiating with respect to time and substituting with Eq. 1 yields:

2 $$\begin{array}{rcl}\frac{d{\boldsymbol{m}}(t)}{dt} & = & \frac{d\delta }{dt}\,m\,\sin (\theta )\hat{{\boldsymbol{e}}}\\ \gamma {\boldsymbol{m}}(t)\times {\boldsymbol{B}}(t) & = & \frac{d\delta }{dt}\,m\,\sin (\theta )\hat{{\boldsymbol{e}}}\\ \gamma m\,B(t)\,\sin (\theta )\hat{{\boldsymbol{e}}} & = & \frac{d\delta }{dt}\,m\,\sin (\theta )\hat{{\boldsymbol{e}}}\,\Rightarrow \,\gamma B(t)=\frac{d\delta }{dt}\end{array}$$

We have retrieved here this elementary demonstration from fundamental magnetodynamics to emphasize that the time-derivative of the precession phase, $\frac{d\delta }{dt}$ (Larmor frequency), is directly proportional to the applied MF even if the latter is time-dependent. Indeed, the assumptions underlying the previous demonstration are that (a) *the direction* of the MF does not change with time (i.e., the DC and the AC components of ***B***(*t*) are parallel, ***B***_*DC*_∥***B***_*AC*_), (b) the amplitude *m* of the magnetic moment is constant, and (c) the angle *θ* between ***B***(*t*) and ***m***(*t*) is also constant. Hence, no constraint applies to *the amplitude* of the MF and so the *instantaneous* Larmor frequency, *ω*_*L*_(*t*), can be written as:

3 $${\omega }_{L}(t)\equiv \frac{d\delta }{dt}=\gamma B(t)=\gamma [{B}_{DC}+{B}_{AC}(t)]$$

Therefore, let us emphasize the fact that when a DC MF is combined with an AC one, Larmor frequency becomes a function of time. While this dependence is negligible when the amplitude of the AC field (${B}_{AC}^{\circ }$) is much smaller than that of the DC field, i.e., when ${B}_{DC}\gg {B}_{AC}^{\circ }$, it does become important when the fields are of the same order, ${B}_{DC} \sim {B}_{AC}^{\circ }$. When the AC field is sinusoidal, by definition, its frequency will be constant, while that of Larmor’s will not. This situation leads to a permanent incoherence between the precessing magnetic moment and the externally applied AC field; meaning that their relative phase will also be time dependent. The key idea proposed here is that it is worth looking at a *frequency modulation* of the AC field, such that the phase change per unit time of the applied AC field is, at all times, identical to that of the precession of the magnetic moment to which the field was tuned considering *γ*. In other words, applied AC field and precession of the ‘engaged’ magnetic moment are phase-locked.

Now, let us make the AC MF be:

4 $${B}_{AC}(t)={B}_{AC}^{\circ }\,{\sin }\,[\varphi \,(t)]$$

where *ϕ*(*t*) is its instantaneous phase, the time-derivative of which gives the instantaneous frequency of *B*_*AC*_(*t*):

5 $${\omega }_{AC}(t)=\varphi ^{\prime} (t)=\frac{d\varphi }{dt}$$

It is immediate to see that if *ϕ*(*t*) = *ωt*, then the instantaneous frequency *ω*_*AC*_(*t*) = *ω* = *constant*, which corresponds to the case of a pure sine. In all other cases, we do not have a pure sine. In order to find the desired modulation, we look into the following identity, which represents the locking of the applied AC MF with the phase changes it produces in the precession of a given magnetic moment:

6 $${\omega }_{AC}(t)={\omega }_{L}(t)$$

We substitute with Eqs 5 and 3 in Eq. 6 to get:

7 $$\frac{d\varphi }{dt}=\frac{d\delta }{dt}$$

8 $$\frac{d\varphi }{dt}=\gamma [{B}_{DC}+{B}_{AC}(t)]$$

We now substitute with Eq. 4:

9 $$\frac{d\varphi }{dt}=\gamma \{{B}_{DC}+{B}_{AC}^{\circ }sin\,[\varphi \,(t)]\}$$

And considering the ratio of AC peak amplitude to DC amplitude to be $\zeta =\frac{{B}_{AC}^{\circ }}{{B}_{DC}}$ yields:

10 $$\frac{d\varphi }{dt}=\gamma {B}_{AC}^{\circ }\{{\zeta }^{-1}+sin\,[\varphi \,(t)]\}$$

And so:

11 $$\frac{d\varphi }{{\zeta }^{-1}+sin\,[\varphi (t)]}=\gamma {B}_{AC}^{\circ }dt$$

We integrate both sides of the equation, where the left hand side can be found in standard tables of integrals^16^ and, for *ζ* < 1, we find that:

12 $$\frac{2\,ta{n}^{-1}\{\frac{{\zeta }^{-1}\,tan[\frac{\varphi (t)}{2}]+1}{\sqrt{{\zeta }^{-2}-1}}\}}{\sqrt{{\zeta }^{-2}-1}}=\gamma {B}_{AC}^{\circ }t+C$$

where *C* is the integration constant, which accounts for the initial conditions (determined by the phase at *t* zero, *ϕ*(*t* = 0)). For simplicity we make *C* = 0 here and, after standard manipulation, the equation is solved explicitly for *ϕ*(*t*):

13 $$\varphi \,(t)=2\,ta{n}^{-1}\{\sqrt{1-{\zeta }^{2}}\,tan[(\frac{\gamma {B}_{AC}^{\circ }}{2}\sqrt{{\zeta }^{-2}-1})t]-\zeta \}$$

And substituting Eq. 13 in Eq. 4 yields:

14 $${B}_{AC}^{PLFM}(t)={B}_{AC}^{\circ }\,{\sin }\{2\,{ta}{{n}}^{-1}\{\sqrt{1-{\zeta }^{2}}\,{\tan }[(\frac{\gamma {B}_{AC}^{\circ }}{2}\sqrt{{\zeta }^{-2}-1})t]-\zeta \}\}$$

Equation 14 represents the main result of this communication, where it must be noted that it is only valid for *ζ* < 1, i.e., ${B}_{AC}^{\circ } < {B}_{DC}$. The case for ${B}_{AC}^{\circ } > {B}_{DC}$ also has an analytical solution but it is not an explicit one, hence it needs to be approached numerically. This case is out of the scope of this work and is currently underway for a future article.

**Figure 2:**
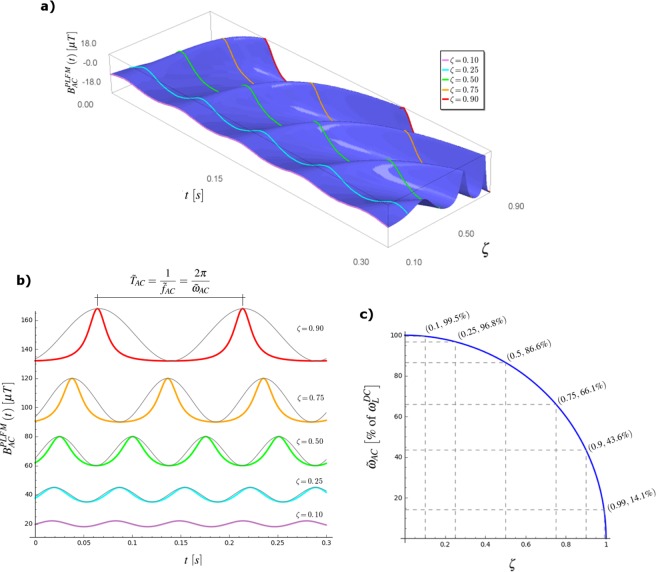
(**a**) 3D plot of the PLFM signals as a function of time and the AC-to-DC ratio (*ζ*), using the gyromagnetic ratio of *Ca*^2 +^, ${\gamma }_{C{a}^{2+}}=4.813\,rad\,{s}^{-1}\,\mu {{\rm{T}}}^{-1}$, and a *B*_*DC*_ = 20 *μ*T. (**b**) PLFM signals for several values of *ζ* (shifted up in the *B*_*AC*_ axis for the sake of visualization). The grey lines are the corresponding sinusoids (of frequency ${\tilde{\omega }}_{AC}$, see Eq. 17), plotted for comparison: it is clear how the PLFM signals depart more and more from a pure sine as *ζ* tends to 1, i.e, as ${B}_{AC}^{\circ }$ approaches *B*_*DC*_. (**c**) The frequency ${\tilde{\omega }}_{AC}$ decreases slowly for “small” *ζ* and rapidly when *ζ* approaches 1.

While Fig. 2a shows a 3D view of how PLFM signals change with time and *ζ*, in Fig. 2b it can be seen that in order to retain the phase-locked condition, the waveform of the AC MF must depart from a sinusoidal shape as *ζ* increases. Besides the distortion from a pure sine, Fig. 2b shows that in spite of the fact that the angular frequency of the AC MF changes permanently (which is why we call it instantaneous), the PLFM signals display a fundamental harmonic, ${\tilde{\omega }}_{AC}$, which dictates the repetition rate of its peaks $({\tilde{f}}_{AC}=\frac{{\tilde{\omega }}_{AC}}{2\pi })$ and, out of inspection of Eq. 14, we note it is given by:

15 $${\tilde{\omega }}_{AC}=\gamma \,{B}_{AC}^{\circ }\sqrt{{\zeta }^{-2}-1},$$

16 $${\tilde{\omega }}_{AC}=\gamma \,\sqrt{{B}_{DC}^{2}-{B}_{AC}^{{\circ }^{2}}},$$

or

17 $${\tilde{\omega }}_{AC}=\gamma \,{B}_{DC}\sqrt{1-{\zeta }^{2}}={\omega }_{L}^{DC}\sqrt{1-{\zeta }^{2}}$$

where ${\omega }_{L}^{DC}=\gamma {B}_{DC}$ is the Larmor frequency due to the DC MF alone. It is evident that ${\tilde{\omega }}_{AC}$ decreases as *ζ* increases. Figure 2c displays how the necessary frequency for phase locking is almost equal to the Larmor frequency (${\omega }_{L}^{DC}$) for small *ζ* (e.g., $\zeta =0.25\,\to \,{\tilde{\omega }}_{AC}=0.968\,{\omega }_{L}^{DC}$), while it is substantially smaller when *ζ* → 1 (e.g., $\zeta =0.9\,\to \,{\tilde{\omega }}_{AC}=0.436\,{\omega }_{L}^{DC}$). Let us refer to ${\tilde{\omega }}_{AC}$ as “Larmor frequency adjusted by the AC MF” or, shortly, “adjusted Larmor frequency” (ALF).

In Supplementary Fig. S1 (animated GIF file, online), phasor diagrams allow to see how the phase difference between the applied MF and the target magnetic moment (a) goes as ${\omega }_{L}^{DC}t$ for *B*_*AC*_ = 0, (b) oscillates with frequency ${\omega }_{L}^{DC}$ when ${B}_{AC}(t)={B}_{AC}^{\circ }\,sin({\omega }_{L}^{DC}t)$ (i.e., the target magnetic moment “follows” the applied MF incoherently), or (c) remains fixed at all times upon a PLFM signal (i.e., the target magnetic moment “follows” the applied MF coherently, phases are locked). It is of note that in this animation the values for *ζ* (0.1, 0.484, 0.866, 0.968, and 0.992) were chosen to the sole effect that it, built as an infinite loop of a finite number of frames, would not have “glitches”. In other words, the values for *ζ* are such that all diagrams display an integer number of complete turns in the generated sequence of frames. Hence, the choice of those values was merely made for visualization purposes: they are not posed to be of any particular (bio)physical relevance.

## Discussion

While the derivation of the PLFM signals is rather straightforward, the extension of its validity is not trivial and deserves to be discussed. There is a key relation upon which the whole derivation rests, namely, that of Eq. 3 which, in turn, holds as long as the angle between the MF and the precessing magnetic moment remains fixed (or changes adiabatically upon small/slow enough perturbations). As mentioned above, the physical interpretation of that geometric constraint is that no damping (i.e., relaxations) can occur during the action of the PLFM signals. In a less rigorous (but more realizable) case, damping and PLFM signals could be compatible if relaxation times were long enough compared to the period of the PLFM signals. Then, PLFM signals would have “enough time to act” before phase-locking is broken. As candidate systems to fulfil this condition, we propose here liquids and viscous media, since they can display relaxation times (T_1_ and T_2_) of up to several seconds. For instance, Ganssle *et al*.^17^ reported a T_1_ = 3.27 s and a T_2_ = 2.08 s for water at 50 *μ*T and 37 °C. Remarkably, these conditions are of paramount relevance in *in vitro* magnetobiology, where cellular cultures are typically grown at that temperature and fields are of the same order. In contrast, solid state systems in general and in particular ferromagnets, which display stronger couplings and much faster magnetodynamics, will probably be inadequate for testing PLFM signals. Another possible scenario where “slow” dynamics could allow an actual locking of the precession phase could be an enzyme cavity with an ion trapped inside. In fact, the dynamics of a nuclear and electronic spinless ion (which develops a magnetic moment inside a protein cavity purely due to its mass and charge being subjected to a central force, $\gamma =\frac{q}{2m}$) could be the target for PLFM signals. An extensively studied example of this is the calcium ion and its binding to Calmodulin (CaM), a key player in the transduction of calcium signals into living cells^18^. Indeed, it has been proven^19^ that the calcium ion can remain inside CaM for intervals in the order of ~1 s: PLFM signals could affect binding times which in turn would affect reaction constants of biological relevance. It must be noted that these ideas have been in the literature for decades^3,7,8,10–12^; thus, the contribution made in this work limits itself to the proposal of a novel frequency modulation, and not of a novel mechanism of interaction.

A distinctive feature of the PLFM signals is that field amplitudes and the waveform (shape and fundamental harmonic) cannot be chosen independently: the choice of DC and AC absolute amplitudes (and not only their ratio, *ζ*) uniquely defines the frequency (the ALF, ${\tilde{\omega }}_{AC}$) and also a degree of distortion from a pure sine. Hence, with PLFM signals three parameters are candidates to be screened as independent variables in systematic studies: *B*_*DC*_, ${B}_{AC}^{\circ }$, and the gyromagnetic ratio *γ*, which does not longer have a role algebraically symmetrical (equivalent) to that of *B*_*DC*_, as it does in the formula ${\omega }_{L}^{DC}=\gamma {B}_{DC}$.

Figure 2b shows that as the amplitude of the AC MF approaches that of the DC MF (${B}_{AC}^{\circ }\to {B}_{DC}$), the valleys of *B*_*AC*_(*t*) become deeper and flatter, which corresponds to instantaneous frequencies approaching zero at the bottom. Reciprocally, the peaks become higher and sharper, meaning that the precession goes from super-slow (almost dwelling) to its maximum (almost $2{\omega }_{L}^{DC}$) in shorter and shorter periods of time (i.e., maximum angular acceleration increases as *ζ* → 1). Since this behaviour clearly departs from that upon sinusoids, it would be of interest to explore how models of reactivity based on the alteration of the dynamics of precession, such as the one by Muehsam and Pilla^12^, or that of Binhi and Prato^14^ would work when PLFM signals are plucked into them (instead of sinusoids).

Interestingly, within a quantum interference model of interaction, Binhi^20,21^ developed a formula that enables to predict the maximally effective parameters of a sequence of square pulses of MF combined with a parallel DC component. The formula relates the DC and AC amplitudes, pulse duration, repetition rate and the gyromagnetic ratio, and is derived from the condition of the MF pulses fully compensating for the precession produced by the DC MF. In contrast, the formula obtained in this work derives from assuming a phase locking between applied AC and engaged magnetic moment: no interaction mechanism has been posed in this work, only the derivation of a class of frequency-modulated signals. In other words, while within his model Binhi *demonstrates* a maximally effective class of square-pulse sequences, here we *hypothesize* that the derived class of PLFM signals are more effective than the corresponding sinusoids (same frequency and amplitude, grey lines in Fig. 2b).

From the experimental point of view, it is at least suggestive to note that experiments in which an effect is seen at extremely low fields but gradually disappears as the field amplitude grows^22,23^ are compatible with the hypothesis that the origin of the effect involves the coherence between the applied AC MF and the Larmor precession induced by the instantaneous DC + AC MF. These experiments fit the following picture: the effect disappears because coherence is lost due to the fact that utilized signals remain at a constant frequency and shape (sinusoidal), instead of decreasing their frequency (to adopt the ALF) and departing from a perfect sine. Were this experiments to show that PLFM signals retain the observed effect for greater amplitudes of the AC field, they would represent a strong indication of a Larmor precession underlying the observed phenomenon. The experiments in question could be a particularly appropriate setting for the test of PLFM signals for at least two reasons: (1) There is a clear uncertainty as to which is the underlying mechanism, and (2) unlike in most of the literature on weak ELF-MF, one of those experiments^22^ has been reproduced by independent laboratories^24–26^. Therefore, if any interesting results were reported on the comparison between sinusoidal and PLFM signals, there would be a relatively high chance of other researchers trying to replicate them.

Lastly, while PLFM signals were derived for AC parallel to DC, it is hypothesized here that for a sufficiently small perpendicular component, ${B}_{A{C}_{\perp }}^{PLFM}\ll {B}_{A{C}_{\parallel }}^{PLFM}$, it might be interesting to explore the non-perpendicular case in which the AC component perpendicular to the DC one is also phase-locked to the precessing magnetic moment:

18 $${B}_{A{C}_{\parallel }}^{PLFM}(t)={B}_{A{C}_{\parallel }}^{\circ }\,{\sin }\,[\varphi (t)]$$

19 $${B}_{A{C}_{\perp }}^{PLFM}(t)={B}_{A{C}_{\perp }}^{\circ }\,{\sin }\,[\varphi (t)]$$

where

20 $$\varphi (t)=2\,{ta}{{n}}^{-1}\{\sqrt{1-{\zeta }^{2}}\,{\tan }\,[(\frac{\gamma {B}_{A{C}_{\parallel }}^{\circ }}{2}\sqrt{{\zeta }^{-2}-1})t]-\zeta \}$$

where $\zeta =\frac{{B}_{A{C}_{\parallel }}^{\circ }}{{B}_{DC}}$ (notice that the time-varying phase, *ϕ*(*t*) is identical for both components). This case is of practical interest because in an actual experimental setting, the slightest imperfection in the parallelism of the AC and DC fields will imply the appearance of that “orthogonal whisper”. The coherence between ${B}_{A{C}_{\perp }}^{PLFM}$ and the magnetic moment precession is analogue to the one commonly achieved in high-field nuclear magnetic resonance (NMR) by using “small” sinusoidal signals at the Larmor frequency. Therefore, the use of PLFM signals could be of interest not only for magnetobiology, but also within the realms of ultra-low field NMR (ULF-NMR), a relatively young discipline^27,28^ thoroughly discussed by Kraus *et al*. in their book^29^. In ULF-NMR, resonance is studied with DC fields in the *μ*T to mT range (as opposed to the Tesla range utilized in standard high field NMR). We suggest here that it might be worth searching for frequency modulations to accomplish coherence in the case of ${B}_{A{C}_{\perp }} \sim {B}_{A{C}_{\parallel }}$, or even ${B}_{A{C}_{\perp }} > {B}_{A{C}_{\parallel }}$. This would imply the task of solving a vector version of what was solved here, which was possible to solve scalarly thanks to the parallelism assumed for the DC and AC fields.

## Supplementary information

Fig-S1_Animated-GIF

Sage-code-for-generating-Fig2-and-animated-GIF-FigS1

## Data Availability

The Sage^30^ code used for generating Fig. 2 and the animated Supplementary Fig. S1 (which also demanded use of the Gimp image manipulation package)^31^ are available as online supplementary materials.
