# Supplementary figures and images for: A new class of signals for magnetobiology research
Source: Sci Rep. 2019 May 16;9:7478. doi: 10.1038/s41598-019-43984-z (PMC6522507; doi:10.1038/s41598-019-43984-z)

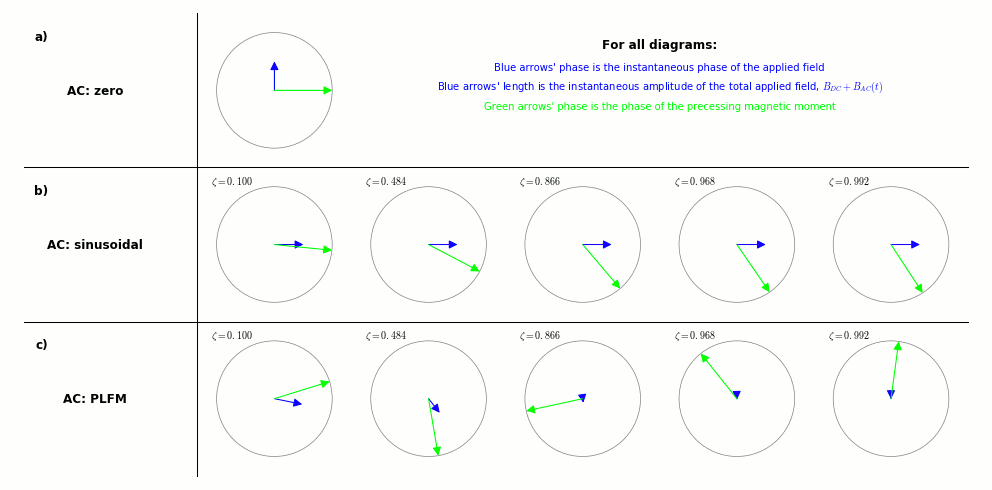

Supplement: Supplementary file 1 — Fig-S1_Animated-GIF [file 41598_2019_43984_MOESM1_ESM.gif]

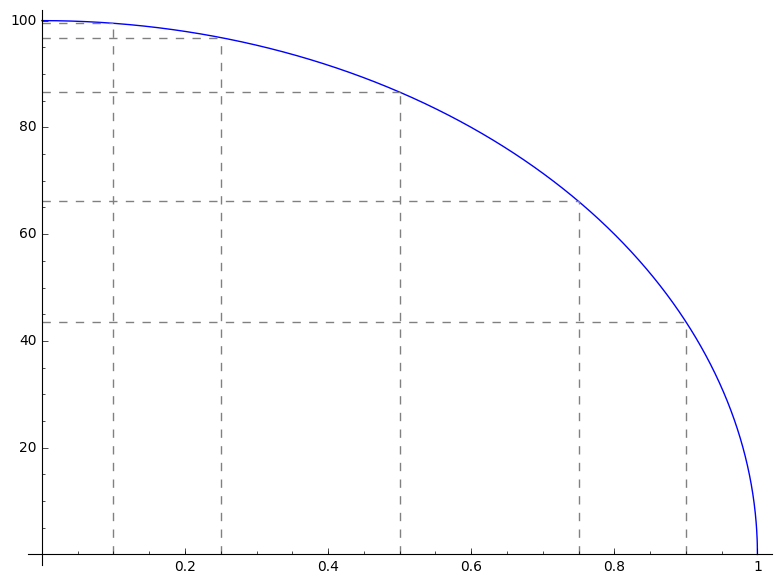

Supplement: Supplementary file 2 — Sage-code-for-generating-Fig2-and-animated-GIF-FigS1 [file 41598_2019_43984_MOESM2_ESM.sws › sage_worksheet/cells/38/sage0.png]

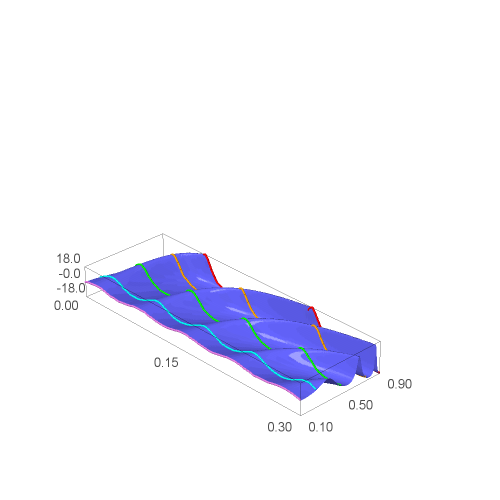

Supplement: Supplementary file 2 — Sage-code-for-generating-Fig2-and-animated-GIF-FigS1 [file 41598_2019_43984_MOESM2_ESM.sws › sage_worksheet/cells/47/.jmol_images/sage0-size500.jmol.png]

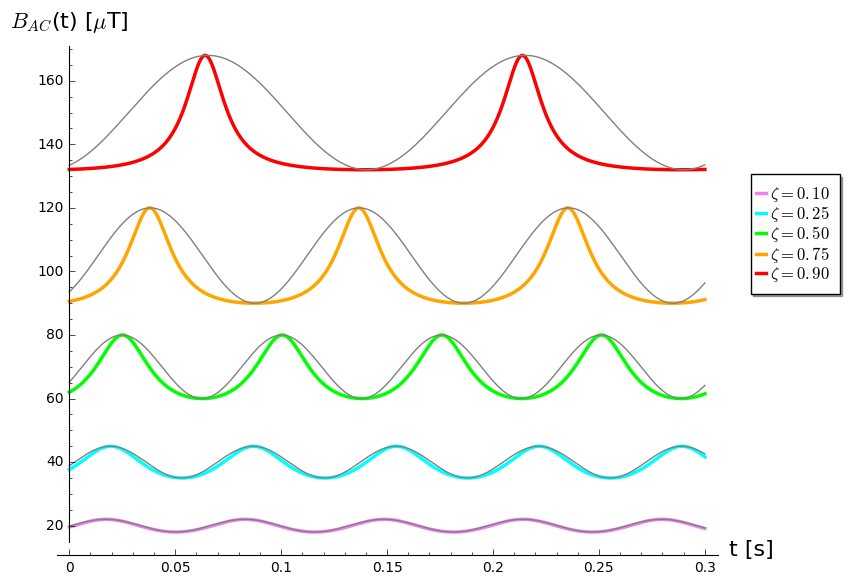

Supplement: Supplementary file 2 — Sage-code-for-generating-Fig2-and-animated-GIF-FigS1 [file 41598_2019_43984_MOESM2_ESM.sws › sage_worksheet/cells/48/sage0.png]
