# Supplementary material for: A new class of signals for magnetobiology research
Source: Sci Rep. 2019 May 16;9:7478. doi: 10.1038/s41598-019-43984-z (PMC6522507; doi:10.1038/s41598-019-43984-z)
Supplement: Supplementary file 2 — Sage-code-for-generating-Fig2-and-animated-GIF-FigS1 [file 41598_2019_43984_MOESM2_ESM.sws › sage_worksheet/worksheet.html]

{{{id=47|
# All codes in this worksheet were authored by Leonardo Makinistian (INFAP, UNSL, CONICET, Argentina) | lmakinistian@gmail.com
#### Code for generating Figure 1 a) ####
gamma = 0.766\*2\*pi # rad/s/microT for Ca+2 ion
t,BDC,ratio = var('t,BDC,ratio') # ratio = BACpeak/BDC
BAC = function('BAC')(t,BDC,ratio)
BAC = BDC\*ratio\*sin(2\*atan(sqrt(1-ratio^2)\*tan(gamma\*BDC\*ratio\*t\*sqrt(1/ratio^2-1)/2)-ratio))
P = plot3d(BAC(BDC=20),(t, 0,0.3),(ratio,0.1,0.9), frame\_aspect\_ratio=[3, 1, 1/3],plot\_points=(400,400))
Param01 = parametric\_plot3d([t,0.1,BAC(BDC=20,ratio=0.1)], (t, 0,0.3), color='violet',thickness=4)
Param025 = parametric\_plot3d([t,0.25,BAC(BDC=20,ratio=0.25)], (t, 0,0.3), color='cyan',thickness=4)
Param05 = parametric\_plot3d([t,0.5,BAC(BDC=20,ratio=0.5)], (t, 0,0.3), color='lime',thickness=4)
Param075 = parametric\_plot3d([t,0.75,BAC(BDC=20,ratio=0.75)], (t, 0,0.3), color='orange',thickness=4)
Param09 = parametric\_plot3d([t,0.9,BAC(BDC=20,ratio=0.9)], (t, 0,0.3), color='red',thickness=4)
Oltu = P+Param01+Param025+Param05+Param075+Param09
show(Oltu)
Oltu.save('c:/Users/lmaki/Desktop/2018\_Paper\_PhaseLock/Fig1a-just.png', figsize = 10)
///
}}}
{{{id=48|
#### Code for generating Figure 1 b) ####
gamma = 0.766\*2\*pi # rad/s/microT for Ca+2 ion
t,BDC,ratio = var('t,BDC,ratio') # ratio = BACpeak/BDC
BAC = function('BAC')(t,BDC,ratio)
BAC = BDC\*ratio\*sin(2\*atan(sqrt(1-ratio^2)\*tan(gamma\*BDC\*ratio\*t\*sqrt(1/ratio^2-1)/2)-ratio))
omega = gamma\*20\*ratio\*sqrt(1/ratio^2-1)
sines = plot(20\*0.1\*sin(omega(BACpeak=20\*ratio,ratio=0.1)\*t+6.20)+20,t,0,0.3,color='grey')+\
plot(20\*0.25\*sin(omega(BACpeak=20\*ratio,ratio=0.25)\*t+6.05)+40,t,0,0.3,color='grey')+\
plot(20\*0.5\*sin(omega(BACpeak=20\*ratio,ratio=0.5)\*t+5.78)+70,t,0,0.3,color='grey')+\
plot(20\*0.75\*sin(omega(BACpeak=20\*ratio,ratio=0.75)\*t+5.42)+105,t,0,0.3,color='grey')+\
plot(20\*0.9\*sin(omega(BACpeak=20\*ratio,ratio=0.9)\*t+5.11)+150,t,0,0.3,color='grey')
BACplot = plot((BAC(BDC=20,ratio=0.1)+20,BAC(BDC=20,ratio=0.25)+40,BAC(BDC=20,ratio=0.5)+70,BAC(BDC=20,ratio=0.75)+105,BAC(BDC=20,ratio=0.9)+150),t,0,0.3,thickness=2.5,\
legend\_label=('$\zeta =0.10$','$\zeta =0.25$','$\zeta =0.50$','$\zeta =0.75$','$\zeta =0.90$',),\
axes\_labels=['t [s]','$B\_{AC}$(t) [$\mu$T]'],color=('violet','cyan','lime','orange','red'), frame=False)+sines;
BACplot.set\_legend\_options(loc=(1.05,0.5))
BACplot.set\_aspect\_ratio(0.0015)
BACplot.show()
BACplot.save('c:/Users/lmaki/Desktop/2018\_Paper\_PhaseLock/Fig1b-just.png')
///
}}}
{{{id=38|
#### Code for generating Figure 1 c) ####
Percent = function('Percent')(ratio)
Percent = (sqrt(1-ratio^2))\*100
P = plot(Percent,ratio,0,1); P.set\_aspect\_ratio=(0.1)
lines = line([(0.1,0), (0.1,Percent(0.1))],color='grey',linestyle='dashed')+\
line([(0.25,0), (0.25,Percent(0.25))],color='grey',linestyle='dashed')+\
line([(0.5,0), (0.5,Percent(0.5))],color='grey',linestyle='dashed')+\
line([(0.75,0), (0.75,Percent(0.75))],color='grey',linestyle='dashed')+\
line([(0.9,0), (0.9,Percent(0.9))],color='grey',linestyle='dashed')+\
line([(0,Percent(0.1)), (0.1,Percent(0.1))],color='grey',linestyle='dashed')+\
line([(0,Percent(0.25)), (0.25,Percent(0.25))],color='grey',linestyle='dashed')+\
line([(0,Percent(0.5)), (0.5,Percent(0.5))],color='grey',linestyle='dashed')+\
line([(0,Percent(0.75)), (0.75,Percent(0.75))],color='grey',linestyle='dashed')+\
line([(0,Percent(0.9)), (0.9,Percent(0.9))],color='grey',linestyle='dashed')
Oltu = P + lines
#Oltu.show().set\_aspect\_ratio=(1.5)
Oltu.show()
print("ratio = 0.1: Percent = %.2f" % round(Percent(0.1),1))
print("ratio = 0.25: Percent = %.2f" % round(Percent(0.25),1))
print("ratio = 0.5: Percent = %.2f" % round(Percent(0.5),1))
print("ratio = 0.75: Percent = %.2f" % round(Percent(0.75),1))
print("ratio = 0.9: Percent = %.2f" % round(Percent(0.9),1))
print("ratio = 0.99: Percent = %.2f" % round(Percent(0.99),1))
///
ratio = 0.1: Percent = 99.50
ratio = 0.25: Percent = 96.80
ratio = 0.5: Percent = 86.60
ratio = 0.75: Percent = 66.10
ratio = 0.9: Percent = 43.60
ratio = 0.99: Percent = 14.10
}}}
{{{id=42|
#### Code for generating Online Supplementary Figure S1 ####
#### This code generates the 200 frames that make up the animation, which was generated by opening the 200 frames (as layers)
#### with the image manipulation software GIMP, optimizing for gif, and saving them as an animated gif file.
from sage.plot.circle import Circle
radius = 1.5
sep = 4
th = 1.5
a = 3.1
Texts = text("AC: zero",(-a\*radius,sep), color = 'black',fontsize='x-large', fontweight = 'bold') +\
text("AC: sinusoidal",(-a\*radius,0), color = 'black',fontsize='x-large', fontweight = 'bold') +\
text("AC: PLFM",(-a\*radius,-sep), color = 'black',fontsize='x-large', fontweight = 'bold')+\
text('$\zeta=0.100$',(-1.1,1.65),fontsize='large', color = 'black')+\
text('$\zeta=0.100$',(-1.1,-2.35),fontsize='large', color = 'black')+\
text('$\zeta=0.484$',(-1.1+sep,1.65),fontsize='large', color = 'black')+\
text('$\zeta=0.484$',(-1.1+sep,-2.35),fontsize='large', color = 'black')+\
text('$\zeta=0.866$',(-1.1+sep\*2,1.65),fontsize='large', color = 'black')+\
text('$\zeta=0.866$',(-1.1+sep\*2,-2.35),fontsize='large', color = 'black')+\
text('$\zeta=0.968$',(-1.1+sep\*3,1.65),fontsize='large', color = 'black')+\
text('$\zeta=0.968$',(-1.1+sep\*3,-2.35),fontsize='large', color = 'black')+\
text('$\zeta=0.992$',(-1.1+sep\*4,1.65),fontsize='large', color = 'black')+\
text('$\zeta=0.992$',(-1.1+sep\*4,-2.35),fontsize='large', color = 'black')+\
text("a)",(-a\*radius\*1.3,sep+sep\*.35), color = 'black',fontsize='x-large', fontweight = 'bold') +\
text("b)",(-a\*radius\*1.3,0+sep\*.35), color = 'black',fontsize='x-large', fontweight = 'bold') +\
text("c)",(-a\*radius\*1.3,-sep+sep\*.35), color = 'black',fontsize='x-large', fontweight = 'bold')+\
text("For all diagrams:",(10,5.2), color = 'black',fontsize='x-large', fontweight = 'bold')+\
text("Blue arrows\' phase is the instantaneous phase of the applied field",(10,5.1-.5\*1), color = 'blue',fontsize='large')+\
text('Blue arrows\' length is the instantaneous amplitude of the total applied field, $B\_{DC}+B\_{AC}(t)$',(10,5.1-.5\*2), color = 'blue',fontsize='large')+\
text("Green arrows\' phase is the phase of the precessing magnetic moment",(10,5.1-.5\*3), rgbcolor = (0,1,0),fontsize='large')
Lines = line([(-radius-0.5,sep+radius+0.5),(-radius-0.5,-sep-radius-0.5)], color = 'black', thickness = th) + line([(-4\*radius-0.5,radius+.5),(4\*sep+radius+.5,radius+.5)], color = 'black', thickness = th) + line([(-4\*radius-0.5,-radius-.5),(4\*sep+radius+.5,-radius-.5)], color = 'black', thickness = th)
Circs = circle((0,sep),radius, color='grey')+circle((0,0),radius, color='grey')+circle((sep,0),radius, color='grey')+\
circle((2\*sep,0),radius, color='grey')+circle((3\*sep,0),radius, color='grey')+circle((4\*sep,0),radius, color='grey')+\
circle((0,-sep),radius, color='grey')+circle((sep,-sep),radius, color='grey')+circle((2\*sep,-sep),radius, color='grey')+\
circle((3\*sep,-sep),radius, color='grey')+circle((4\*sep,-sep),radius, color='grey')
gamma = 0.766\*2\*pi # rad/s/microT for Ca+2 ion
BDC = 0.75 # microTeslas
omegaLDC = gamma\*BDC
ratios = [0.1,0.484,0.866,0.968,0.992]
t,ratio = var('t,ratio')
BAC = function('BAC')(t,ratio)
BAC = BDC\*ratio\*sin(2\*atan(sqrt(1-ratio^2)\*tan(gamma\*BDC\*ratio\*t\*sqrt(1/ratio^2-1)/2)-ratio))
phaseFM = function('BAC')(t,ratio)
phaseFM = 2\*atan(sqrt(1-ratio^2)\*tan(gamma\*BDC\*ratio\*t\*sqrt(1/ratio^2-1)/2)-ratio)
Engaged = function('Engaged')(ratio,t)
T = 1.74\*8
N = 25\*8
delta = T/N
awidth = 1.5
asize = 3.5
DCfield = arrow((0,+sep), (0,+sep+0.75), rgbcolor = (0, 0, 1), width = awidth, arrowsize = asize)
NullAC = [arrow((0,+sep), (1.5\*cos(omegaLDC\*time),sep+1.5\*sin(omegaLDC\*time)), rgbcolor = (0, 1, 0), width = awidth, arrowsize = asize) for time in srange(0,T,delta)]
#######################################
#Second (center) floor of the graphic:
AppliedSine01 = [arrow((0,0), ((BDC + ratios[0]\*BDC\*sin(omegaLDC\*time))\*cos(omegaLDC\*time),(BDC + ratios[0]\*BDC\*sin(omegaLDC\*time))\*sin(omegaLDC\*time)), width = awidth, arrowsize = asize) for time in srange(0,T,delta)]
AppliedSine02 = [arrow((sep,0), (sep+(BDC + ratios[1]\*BDC\*sin(omegaLDC\*time))\*cos(omegaLDC\*time),(BDC + ratios[1]\*BDC\*sin(omegaLDC\*time))\*sin(omegaLDC\*time)), width = awidth, arrowsize = asize) for time in srange(0,T,delta)]
AppliedSine03 = [arrow((2\*sep,0), (2\*sep+(BDC + ratios[2]\*BDC\*sin(omegaLDC\*time))\*cos(omegaLDC\*time),(BDC + ratios[2]\*BDC\*sin(omegaLDC\*time))\*sin(omegaLDC\*time)), width = awidth, arrowsize = asize) for time in srange(0,T,delta)]
AppliedSine04 = [arrow((3\*sep,0), (3\*sep+(BDC + ratios[3]\*BDC\*sin(omegaLDC\*time))\*cos(omegaLDC\*time),(BDC + ratios[3]\*BDC\*sin(omegaLDC\*time))\*sin(omegaLDC\*time)), width = awidth, arrowsize = asize) for time in srange(0,T,delta)]
AppliedSine05 = [arrow((4\*sep,0), (4\*sep+(BDC + ratios[4]\*BDC\*sin(omegaLDC\*time))\*cos(omegaLDC\*time),(BDC + ratios[4]\*BDC\*sin(omegaLDC\*time))\*sin(omegaLDC\*time)), width = awidth, arrowsize = asize) for time in srange(0,T,delta)]
EngagedSine01 = [arrow((0,0), (1.5\*cos(gamma\*0.5\*(BDC\*time - ratios[0]\*BDC\*cos(omegaLDC\*time)/omegaLDC)),1.5\*sin(gamma\*0.5\*(BDC\*time - ratios[0]\*BDC\*cos(omegaLDC\*time)/omegaLDC))), rgbcolor = (0, 1, 0),width = awidth, arrowsize = asize) for time in srange(0,T,delta)]
EngagedSine02 = [arrow((sep,0), (sep+1.5\*cos(gamma\*0.5\*(BDC\*time - ratios[1]\*BDC\*cos(omegaLDC\*time)/omegaLDC)),1.5\*sin(gamma\*0.5\*(BDC\*time - ratios[1]\*BDC\*cos(omegaLDC\*time)/omegaLDC))), rgbcolor = (0, 1, 0), width = awidth, arrowsize = asize) for time in srange(0,T,delta)]
EngagedSine03 = [arrow((2\*sep,0), (2\*sep+1.5\*cos(gamma\*0.5\*(BDC\*time - ratios[2]\*BDC\*cos(omegaLDC\*time)/omegaLDC)),1.5\*sin(gamma\*0.5\*(BDC\*time - ratios[2]\*BDC\*cos(omegaLDC\*time)/omegaLDC))), rgbcolor = (0, 1, 0), width = awidth, arrowsize = asize) for time in srange(0,T,delta)]
EngagedSine04 = [arrow((3\*sep,0), (3\*sep+1.5\*cos(gamma\*0.5\*(BDC\*time - ratios[3]\*BDC\*cos(omegaLDC\*time)/omegaLDC)),1.5\*sin(gamma\*0.5\*(BDC\*time - ratios[3]\*BDC\*cos(omegaLDC\*time)/omegaLDC))), rgbcolor = (0, 1, 0), width = awidth, arrowsize = asize) for time in srange(0,T,delta)]
EngagedSine05 = [arrow((4\*sep,0), (4\*sep+1.5\*cos(gamma\*0.5\*(BDC\*time - ratios[4]\*BDC\*cos(omegaLDC\*time)/omegaLDC)),1.5\*sin(gamma\*0.5\*(BDC\*time - ratios[4]\*BDC\*cos(omegaLDC\*time)/omegaLDC))), rgbcolor = (0, 1, 0), width = awidth, arrowsize = asize) for time in srange(0,T,delta)]
plot(gamma\*0.5\*(BDC + ratios[0]\*BDC\*sin(omegaLDC\*t)), t, 0,T)
#######################################
#Third (botton) floor of the graphic:
AppliedPLFM01 = [arrow((0,-sep), ((BDC+BDC\*ratios[0]\*sin(2\*atan(sqrt(1-ratios[0]^2)\*tan(gamma\*BDC\*ratios[0]\*time\*sqrt(1/ratios[0]^2-1)/2)-ratios[0])))\*cos(2\*atan(sqrt(1-ratios[0]^2)\*tan(gamma\*BDC\*ratios[0]\*time\*sqrt(1/ratios[0]^2-1)/2)-ratios[0])),-sep+(BDC+BDC\*ratios[0]\*sin(2\*atan(sqrt(1-ratios[0]^2)\*tan(gamma\*BDC\*ratios[0]\*time\*sqrt(1/ratios[0]^2-1)/2)-ratios[0])))\*sin(2\*atan(sqrt(1-ratios[0]^2)\*tan(gamma\*BDC\*ratios[0]\*time\*sqrt(1/ratios[0]^2-1)/2)-ratios[0]))), width = awidth, arrowsize = asize) for time in srange(0,T,delta)]
AppliedPLFM02 = [arrow((sep,-sep), (sep+(BDC+BDC\*ratios[1]\*sin(2\*atan(sqrt(1-ratios[1]^2)\*tan(gamma\*BDC\*ratios[1]\*time\*sqrt(1/ratios[1]^2-1)/2)-ratios[1])))\*cos(2\*atan(sqrt(1-ratios[1]^2)\*tan(gamma\*BDC\*ratios[1]\*time\*sqrt(1/ratios[1]^2-1)/2)-ratios[1])),-sep+(BDC+BDC\*ratios[1]\*sin(2\*atan(sqrt(1-ratios[1]^2)\*tan(gamma\*BDC\*ratios[1]\*time\*sqrt(1/ratios[1]^2-1)/2)-ratios[1])))\*sin(2\*atan(sqrt(1-ratios[1]^2)\*tan(gamma\*BDC\*ratios[1]\*time\*sqrt(1/ratios[1]^2-1)/2)-ratios[1]))), width = awidth, arrowsize = asize) for time in srange(0,T,delta)]
AppliedPLFM03 = [arrow((2\*sep,-sep), (2\*sep+(BDC+BDC\*ratios[2]\*sin(2\*atan(sqrt(1-ratios[2]^2)\*tan(gamma\*BDC\*ratios[2]\*time\*sqrt(1/ratios[2]^2-1)/2)-ratios[2])))\*cos(2\*atan(sqrt(1-ratios[2]^2)\*tan(gamma\*BDC\*ratios[2]\*time\*sqrt(1/ratios[2]^2-1)/2)-ratios[2])),-sep+(BDC+BDC\*ratios[2]\*sin(2\*atan(sqrt(1-ratios[2]^2)\*tan(gamma\*BDC\*ratios[2]\*time\*sqrt(1/ratios[2]^2-1)/2)-ratios[2])))\*sin(2\*atan(sqrt(1-ratios[2]^2)\*tan(gamma\*BDC\*ratios[2]\*time\*sqrt(1/ratios[2]^2-1)/2)-ratios[2]))), width = awidth, arrowsize = asize) for time in srange(0,T,delta)]
AppliedPLFM04 = [arrow((3\*sep,-sep), (3\*sep+(BDC+BDC\*ratios[3]\*sin(2\*atan(sqrt(1-ratios[3]^2)\*tan(gamma\*BDC\*ratios[3]\*time\*sqrt(1/ratios[3]^2-1)/2)-ratios[3])))\*cos(2\*atan(sqrt(1-ratios[3]^2)\*tan(gamma\*BDC\*ratios[3]\*time\*sqrt(1/ratios[3]^2-1)/2)-ratios[3])),-sep+(BDC+BDC\*ratios[3]\*sin(2\*atan(sqrt(1-ratios[3]^2)\*tan(gamma\*BDC\*ratios[3]\*time\*sqrt(1/ratios[3]^2-1)/2)-ratios[3])))\*sin(2\*atan(sqrt(1-ratios[3]^2)\*tan(gamma\*BDC\*ratios[3]\*time\*sqrt(1/ratios[3]^2-1)/2)-ratios[3]))), width = awidth, arrowsize = asize) for time in srange(0,T,delta)]
AppliedPLFM05 = [arrow((4\*sep,-sep), (4\*sep+(BDC+BDC\*ratios[4]\*sin(2\*atan(sqrt(1-ratios[4]^2)\*tan(gamma\*BDC\*ratios[4]\*time\*sqrt(1/ratios[4]^2-1)/2)-ratios[4])))\*cos(2\*atan(sqrt(1-ratios[4]^2)\*tan(gamma\*BDC\*ratios[4]\*time\*sqrt(1/ratios[4]^2-1)/2)-ratios[4])),-sep+(BDC+BDC\*ratios[4]\*sin(2\*atan(sqrt(1-ratios[4]^2)\*tan(gamma\*BDC\*ratios[4]\*time\*sqrt(1/ratios[4]^2-1)/2)-ratios[4])))\*sin(2\*atan(sqrt(1-ratios[4]^2)\*tan(gamma\*BDC\*ratios[4]\*time\*sqrt(1/ratios[4]^2-1)/2)-ratios[4]))), width = awidth, arrowsize = asize) for time in srange(0,T,delta)]
EngagedPLFM01 = [arrow((0,-sep), (1.5\*cos(0.5+2\*atan(sqrt(1-ratios[0]^2)\*tan(gamma\*BDC\*ratios[0]\*time\*sqrt(1/ratios[0]^2-1)/2)-ratios[0])),-sep+1.5\*sin(0.5+2\*atan(sqrt(1-ratios[0]^2)\*tan(gamma\*BDC\*ratios[0]\*time\*sqrt(1/ratios[0]^2-1)/2)-ratios[0]))), rgbcolor=(0,1,0), width = awidth, arrowsize = asize) for time in srange(0,T,delta)]
EngagedPLFM02 = [arrow((sep,-sep), (sep+1.5\*cos(-0.5+2\*atan(sqrt(1-ratios[1]^2)\*tan(gamma\*BDC\*ratios[1]\*time\*sqrt(1/ratios[1]^2-1)/2)-ratios[1])),-sep+1.5\*sin(-0.5+2\*atan(sqrt(1-ratios[1]^2)\*tan(gamma\*BDC\*ratios[1]\*time\*sqrt(1/ratios[1]^2-1)/2)-ratios[1]))), rgbcolor=(0,1,0), width = awidth, arrowsize = asize) for time in srange(0,T,delta)]
EngagedPLFM03 = [arrow((2\*sep,-sep), (2\*sep+1.5\*cos(-1.5+2\*atan(sqrt(1-ratios[2]^2)\*tan(gamma\*BDC\*ratios[2]\*time\*sqrt(1/ratios[2]^2-1)/2)-ratios[2])),-sep+1.5\*sin(-1.5+2\*atan(sqrt(1-ratios[2]^2)\*tan(gamma\*BDC\*ratios[2]\*time\*sqrt(1/ratios[2]^2-1)/2)-ratios[2]))), rgbcolor=(0,1,0), width = awidth, arrowsize = asize) for time in srange(0,T,delta)]
EngagedPLFM04 = [arrow((3\*sep,-sep), (3\*sep+1.5\*cos(-2.5+2\*atan(sqrt(1-ratios[3]^2)\*tan(gamma\*BDC\*ratios[3]\*time\*sqrt(1/ratios[3]^2-1)/2)-ratios[3])),-sep+1.5\*sin(-2.5+2\*atan(sqrt(1-ratios[3]^2)\*tan(gamma\*BDC\*ratios[3]\*time\*sqrt(1/ratios[3]^2-1)/2)-ratios[3]))), rgbcolor=(0,1,0), width = awidth, arrowsize = asize) for time in srange(0,T,delta)]
EngagedPLFM05 = [arrow((4\*sep,-sep), (4\*sep+1.5\*cos(3+2\*atan(sqrt(1-ratios[4]^2)\*tan(gamma\*BDC\*ratios[4]\*time\*sqrt(1/ratios[4]^2-1)/2)-ratios[4])),-sep+1.5\*sin(3+2\*atan(sqrt(1-ratios[4]^2)\*tan(gamma\*BDC\*ratios[4]\*time\*sqrt(1/ratios[4]^2-1)/2)-ratios[4]))), rgbcolor=(0,1,0), width = awidth, arrowsize = asize) for time in srange(0,T,delta)]
Allto = [DCfield + Circs + Lines + Texts + NullAC[j] + AppliedSine01[j] + AppliedSine02[j] + AppliedSine03[j] +\
AppliedSine04[j] + AppliedSine05[j] + EngagedSine01[j] + EngagedSine02[j] +\
EngagedSine03[j] + EngagedSine04[j] + EngagedSine05[j] + AppliedPLFM01[j] +\
AppliedPLFM02[j] + AppliedPLFM03[j] + AppliedPLFM04[j] + AppliedPLFM05[j] + EngagedPLFM01[j] + EngagedPLFM02[j] +\
EngagedPLFM03[j] + EngagedPLFM04[j] + EngagedPLFM05[j] for j in srange(len(srange(0,T,delta)))]
for j in srange(len(srange(0,T,delta))):
if j < 10:
prefix = '00'
elif j < 100:
prefix = '0'
else:
prefix = ''
Allto[j].save('c:/Users/lmaki/Desktop/2018\_Paper\_PhaseLock/FrameJUST'+prefix+str(j)+'.png',axes = False,figsize=20,dpi=50)
# Frames are saved as Frame001.png, Frame002.png, ... , Frame199.png
#show(Allto[j],axes = False,figsize=20,dpi=90)
# print j
///
}}}
{{{id=49|
///
}}}
